# Screen-Aware Reverse Tone Mapping

**DOI:** 10.3390/jimaging12060250

**Published:** 2026-06-06

**Authors:** Mihnea-Petrut-Ilie Mitrache, Costin-Anton Boiangiu

**Affiliations:** Faculty of Automatic Control and Computer Science, National University of Science and Technology Politehnica of Bucharest (UPB), 060042 Bucharest, Romania; costin.boiangiu@upb.ro

**Keywords:** HDR imaging, reverse tone mapping, display-aware, neural network, peak brightness, exposure fusion

## Abstract

High dynamic range (HDR) imaging offers an enhanced visual experience by capturing a wider range of real-world luminance levels in digital images. Driven by the increasing demand for high-quality visuals, HDR monitor technology has seen significant advancements. As such monitors become commonplace in both consumer and professional settings, efficient methods are needed for both converting standard dynamic range (SDR) content to HDR—known as reverse tone mapping—and optimizing natural HDR lighting content for display on HDR monitors. A reverse tone mapping procedure aims to produce natural lighting levels, but even on high-end HDR monitors, such images still require adjustment to avoid hard clipping. This paper presents a solution that jointly does both steps: (1) reverse tone mapping to a display-aware HDR representation, and (2) direct generation of an image tailored for a chosen monitor brightness value. We propose a novel neural network architecture conditioned on the target peak brightness via a lightweight multi-layer perceptron (MLP) module injected at the bottleneck, which predicts a bracketed stack of LDR exposures serving as the method’s HDR representation. In this manner, the ill-posed tone mapping problem is guided by auxiliary information about display characteristics, improving visual quality. Experiments throughout the full consumer HDR range (100–4000 nits) show consistent improvements over the display-agnostic baseline in peak luminance utilization, local contrast, color and perceptual quality.

## 1. Introduction

For decades, we were accustomed to displays with limited dynamic range. The well-known tone mapping process was used to map the wide luminance range of real-world scenes to the commonly available standard dynamic range displays. Now, with the advent of high dynamic range technology, we are witnessing a paradigm shift and an increased need to transform SDR content.

HDR displays have revolutionized how we experience digital content by providing a broader luminance range, resulting in more vibrant, realistic images. At the heart of this enhanced visual experience lies the hardware’s peak brightness capability, the highest luminance a monitor can produce. This specification, expressed in nits (candelas per square meter, cd/m^2^), fundamentally shapes the clarity, vibrancy, and realism of displayed content across applications. Variations in peak brightness directly influence how media is perceived, affecting everything from highlight detail to overall contrast. Numerous studies underscore the pivotal role of peak brightness in determining perceived image quality and user experience.

In gaming, high peak brightness is essential for accurately rendering specular highlights, such as glints of sunlight or shiny particle-based effects. The impact is even more pronounced in virtual reality (VR); Chen et al. [1] showed that the user’s sense of immersion and presence relies heavily on the display’s ability to reproduce realistic luminance and contrast ratios. Furthermore, as video streaming adopts efficient codecs like the royalty-free AOMedia Video (AV1), the connection between compression artifacts and display capabilities becomes significant. Hammou et al. [2] indicate that the perceived quality of streamed HDR content is strictly correlated with the peak luminance of the end-user’s device. Consequently, peak brightness is not merely a hardware specification but a decisive variable in the content delivery pipeline.

Despite this rapid advancement in display hardware, the transition to an HDR ecosystem faces a significant challenge: the vast legacy standard dynamic range (SDR) content. Most of the world’s digital imagery—spanning historical archives, cinema, social media, and personal photography—was captured and stored using traditional pipelines with limited luminance range. This creates a distinct gap: modern screens can reach astonishing brightness, yet they are largely fed content designed for the displays of the past. To bridge this gap, we must rely on reverse tone mapping (rTM). This process ideally aims to expand the luminance and contrast of an SDR input to the natural spectrum, effectively “hallucinating” the dynamic range that was lost or compressed during the original capture. Standard approaches would consider just the creation of the HDR image, whose details would either be insufficient or clipped on the final renderer.

We propose a deep-learning-based approach to address the dual challenges of SDR-to-HDR conversion and HDR display optimization. Building on Endo et al. [3], we introduce a novel neural network architecture that not only performs SDR-to-HDR conversion but also incorporates display peak brightness into the optimization process. By integrating it, our model can produce outputs that are not only visually appealing but also tailored to the display device’s specific capabilities. This dual-focus approach addresses the ill-posed nature of reverse tone mapping by providing additional context, thereby improving visual quality and enhancing the immersive viewing experience. The image obtained is screen-ready and does not need any further processing. Our contributions are as follows:

- We design a neural network that integrates target peak brightness into the reconstruction process. Unlike traditional methods, such as that of Endo et al. [3], that target a fixed HDR standard, our model simultaneously performs dynamic range expansion and tone optimization tailored to the target display’s capabilities.
- We demonstrate through experiments that conditioning the network on the display’s peak brightness yields greater colorfulness, local contrast, and overall brightness distribution compared to a baseline monitor-agnostic solution.
- We introduce a display-aware pipeline that generates images for a wide range of HDR monitors by conditioning the full rendering path on the target peak brightness. This approach minimizes clipping and preserves natural image context.

## 2. Related Work

### 2.1. Reverse Tone Mapping

rTM is a research area that has attracted growing interest in the last decade. The concept was first introduced by Banterle et al. [4]. They used a so-called expanded map to identify possible highlights in images within the SDR image, using the median cut algorithm. By applying a global expansion curve derived from the inverse of a standard photographic tone-reproduction operator and modulating it with the expanded map, images with enhanced dynamic range were generated. Other early methods emerged, showcasing diverse rTM operators. All of them relied on handcrafted heuristics to identify and enhance specific image regions. Akyüz et al. [5] concluded that simple operators, even linear expansion, can yield satisfactory results. However, without contextual understanding, these methods often produce artifacts or unnatural appearances.

In the second half of the 2010s, AI-based methods began to gain traction across a range of image processing tasks. rTM was no exception. Operators evolved from simple image pixel processing functions to complex data-dependent models. Eilertsen et al. [6] were among the first to make use of deep neural networks to complete the task. Intuitively called HDRCNN, their network aims to isolate overexposed areas and blend them back into the linearized input image. The overall idea was similar to that of Banterle et al., but the expanded map was learned from data rather than handcrafted. The approach cannot be considered end-to-end, as the linearization step is performed outside the network. Furthermore, it focuses only on recovering overexposed areas, leaving other parts of the image that could benefit from dynamic range expansion out of reach. Cai et al. [7] demonstrated that multi-exposure image stacks can act as effective references for training a single image contrast enhancer, establishing the bracketed-exposure paradigm as a practical deep learning target.

Endo et al. [3] proposed an early and significant deep-learning-based framework to infer an HDR image from a single SDR input. In contrast to previous methods, the goal was to reconstruct the entire HDR image, not just specific areas. The authors designed a novel architecture that predicts multiple exposure levels from the SDR input, combining them in a post-processing step to obtain the final HDR output. This method demonstrates a superior understanding of image brightness distribution. Furthermore, a direct mapping done only in the network would need huge training data and time because of the vast gradient space. This stands as the basis for our work.

The above-mentioned learning-based methods focus solely on the SDR-to-HDR conversion task and are agnostic to display characteristics. The mapping is learned from the overall luminance distribution in the training data. When inference is performed on a mid-range (400 nit) or new-generation (4000 nit) HDR monitor, the results are suboptimal. This is a major limitation, considering the dynamic market of HDR displays.

Martins et al. [8] offer a comprehensive analysis of HDR reconstruction, underscoring the emergence of diffusion-based approaches in this domain. For instance, Bemana et al. [9] synthesize a bracketed stack of LDR exposures via multiple coupled diffusion denoising steps, which are subsequently merged into a single HDR image—a process conceptually aligned with our framework. Alternatively, Goswami et al. [10] (DITMO) leverage diffusion-based inpainting, guided by semantic segmentation, to hallucinate details in saturated regions across exposures. Nevertheless, while these methods are generative and powerful, they all produce display-agnostic HDR outputs that do not account for the target display’s peak brightness.

### 2.2. Display Awareness

The concept of display awareness is more widely explored in the context of tone mapping (HDR-to-SDR conversion). Reinhard et al. [11] introduced a tone mapping operator based on a photographic approach. Dodging and burning techniques were used to enhance local contrast, simulating the practices of professional photographers. Drago et al. [12] constructed upon this work with the goal of adaptability and extensibility to address both the capabilities of displaying methods at that time and their future evolution, arguing that tone mapping efficiency is linked to the brightness of the final display.

Mantiuk et al. [13] discussed the importance of display characteristics in tone mapping. They argued that it is impossible to design a universal tone mapping operator that performs well across all display types. Their method accounts for peak brightness and other display parameters to optimize the compression curve, guaranteeing that the consequent image preserves as much visual information as possible for that specific screen. Eilertsen et al. [14] pursued this direction with a real-time noise-aware tone mapping approach that adapts compression curves to the display’s peak brightness, demonstrating that display-adaptive strategies consistently outperform static operators across diverse monitor configurations.

As stated above, those methods are designed for direct tone mapping, not the reverse process. Most of the existing rTM techniques do not incorporate any target display characteristics into their models. The only prior work specifically addressing this challenge is by Luzardo et al. [15], who introduced a display-adaptive reverse tone mapping (rTM) method. Their approach uses a set of handcrafted rules to adjust the rTM process based on the target display’s peak brightness. However, their method relies on traditional image processing techniques rather than deep learning, limiting its adaptability and performance compared to modern neural-network-based approaches.

To the best of our knowledge, no prior work has explored integrating display awareness into SDR-to-HDR conversion in a deep learning environment. Our novel architecture addresses this gap by proposing a deep-learning-based approach that not only performs SDR-to-HDR conversion but also optimizes the output for specific HDR monitor characteristics, leveraging peak brightness.

## 3. HDR Reconstruction Model

### 3.1. Problem Definition

Reverse tone mapping (rTM) aims to reconstruct a high dynamic range (HDR) image from a standard dynamic range (SDR) input. Formally, given an SDR image I_SDR_, the objective is to learn a mapping function f:

(1) $$I_{HDR}\;=\;f\left(I_{SDR}\right),$$

where I_HDR_ represents the reconstructed HDR image.

This operation is inherently ill-posed because illuminance information is irretrievably lost during SDR quantization and tone mapping. Consequently, no unique solution exists. As previously discussed, nearly all existing deep-learning-based rTM operators rely solely on the SDR input and learn a generic mapping function f derived from the luminance distribution of the training data. We contend that the mapping function f should explicitly depend on the target HDR display characteristics, specifically the peak brightness P_max_. Incorporating this display-specific constraint yields a more well-defined problem formulation.

We redefine the problem as a learning task conditioned on the target display’s peak brightness:

(2) $$I_{HDR}\;=\;f\left(I_{SDR},\;P_{max}\;\right)$$

We strive to generate a set of features that best reconstruct the HDR image for the specific display luminance capabilities. Function f is not a direct mapping, but it provides enough information to generate the HDR output. The whole pipeline will be described step by step below.

### 3.2. Architecture Overview

The proposed architecture builds on Endo et al.’s [3] contribution. It relies on an encoder–decoder backbone to fuse multiple exposure levels predicted from the SDR input. The stack of bracketed exposures is then combined in a post-processing step to obtain the final HDR output. Mertens et al. [16] define a method for merging them into a view of the HDR image. Our key innovation lies in integrating display awareness into this framework. The image generated after merging can be directly output since the creational process considered the display’s peak brightness.

**Figure 1:**
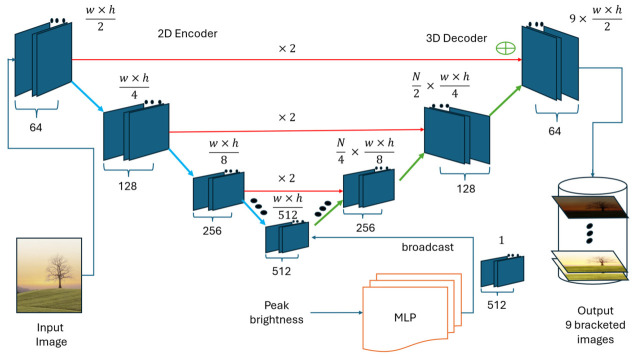
Overview of the proposed display-aware reverse tone mapping architecture. The system receives an SDR input image and a target peak brightness value, which are jointly processed. The peak brightness scalar is introduced at the bottleneck through a lightweight MLP, enabling the decoder to generate nine bracketed LDR exposures, adapted for the specific peak brightness. Blue arrows denote downsampling, green arrows denote upsampling, and red arrows indicate skip connections carrying feature maps relative to the current decoder stage. The ⊕ symbol represents element-wise addition.

The architecture consists of two main modules, which are described below. Figure 1 shows an overview of the proposed architecture. As a glimpse of the whole pipeline, we start with the SDR image which is fed along with the target display scalar brightness into the model. A stack of targeted 9 bracketed LDR exposures is obtained. The value 9 is chosen targeting optimal further reconstruction, based on the dynamic luminance range. These are combined into the final image using a merging scheme described below.

#### 3.2.1. Bracketed Exposure Generator

Our learning problem is best tackled with a deep convolutional neural network (CNN) using a U-Net architecture. Eilertsen et al. [6] demonstrated that the skip connections of the U-Net help retain image details, thereby enhancing the final image quality. While a straightforward autoencoder could establish a one-to-one mapping, reconstructing the HDR image directly from the SDR input, such a method would require substantial training data and computational time due to the large gradient space involved. A better approach is to use an encoder–decoder, with different input and output sizes. The decoder will produce multiple image exposures from the SDR input. It will be composed of:

- 2D Encoder: The encoder processes the SDR input I_SDR through a series of 2D convolutional layers to extract deep semantic features. As in Endo et al. [3], we use a 9-layer encoder that handles 512 × 512 resolution inputs, encoding the image into latent space. At the bottleneck, this information can be further enhanced using display data conditioning and then spatially extended.
- 3D Decoder: Unlike standard image-to-image translation networks, the decoder utilizes 3D deconvolutional layers. This design enables the network to effectively model correlations among different exposure values, ensuring consistency across the predicted bracketed stack.

As mentioned by Endo et al. [3], 3D deconvolutions are mainly used in video processing, with the temporal dimension represented as depth. Here, we repurpose this concept by treating exposure levels as the third dimension. This structure permits the network to learn to consistently reproduce luminance across different encoding levels of the input. This highlights our key idea: HDR reconstruction should be display-aware rather than a generic mapping. How we fit the display data into the network is described in the next section.

The decoder’s design mimics the encoder. Nine layers of 3D deconvolutions are used, with filter sizes set to 512, 512, 512, 512, 512, 256, 128, 64, and finally 3 to output the RGB stack. We apply batch normalization [17] and the leaky ReLU activation function proposed by Maas et al. [18] after every layer except the last. The final layer utilizes a sigmoid activation function to constrain the output pixel values to the valid range [0, 1].

Training the above deep network, which uses 3D deconvolutions, is difficult. Previous work by Brock et al. [19] and Vondrick et al. [20] has shown that temporal instability manifests as blurriness and flickering artifacts. The instability is equally present when treating exposure levels as the temporal dimension. To mitigate, as in Endo et al. [3], we use skip connections. Skip connections are added for the classical U-Net pattern. Residual blocks, proposed by He et al. [21], connect the two parts of the network. The linking is done between layer i of the encoder and layer n−i of the decoder, allowing the utilization of low-level features during the reconstruction process.

A challenge in our architecture is the incompatibility in dimensionality between the modules: the encoder processes 2D tensors (W × H × C), while the decoder operates on 3D tensors (N × W × H × C). To address this, as in Endo et al. [3], we extend along the exposure dimension by repeating the 2D feature maps N times before feeding them into the decoder. This simple yet effective strategy ensures that the rich features extracted by the encoder are fully leveraged during 3D decoding. This backbone architecture is chosen for its versatility and proven efficiency in image-to-image translation tasks, even with reduced training capability. We adapt it to our specific needs by integrating display awareness, as detailed in the next module.

We argue that, although recent work in HDR processing has begun to explore transformer-based architectures, CNNs still offer significant advantages. Transformers are known for their ability to model long-range dependencies, suitable for the large gradient domain, as shown by Wang et al. [22]. CNNs, on the other hand, maintain a distinct advantage in local feature extraction and computational efficiency, revealed in a comparative study by Martins et al. [8], particularly when processing high-resolution exposure stacks. Transformers also demand much more data and time for training in such scenarios.

#### 3.2.2. Display-Aware Module

Our novel contribution lies in integrating display awareness into the HDR reconstruction process. We define this module, which conditions the overall learning process on the target HDR monitor’s peak brightness, P_sample_.

**Figure 2:**
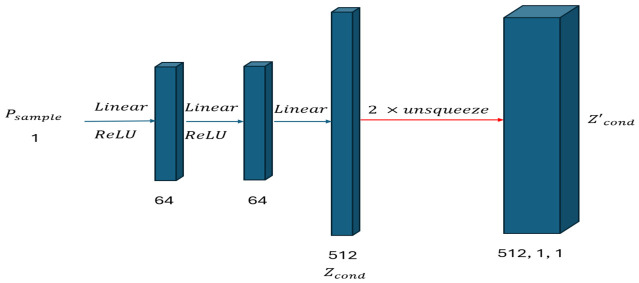
Architecture of the display-aware MLP module. The normalized peak brightness scalar is passed through two successive Linear + ReLU layers, followed by a final linear layer, resulting in a 512-dimensional conditioning vector. This vector is then spatially expanded using two unsqueeze operations to form ${Z^{\prime }}_{cond}$, which is injected at the bottleneck.

Display-aware data encoding is modeled in parallel with the main SDR input encoding logic. The result is injected at the network’s bottleneck to control the decoding process. The 3rd dimension of data in the decoding stage, luminance level, is adjusted across the bracketed exposures. The target display peak brightness is used as a scalar input. The value is normalized to the [0, 1] range, based on the maximum brightness supported by current HDR monitors. The value is then fed into a small fully connected network, an MLP. The MLP consists of three fully connected layers with ReLU activation functions between them. Figure 2 presents the architecture of the MLP module for brightness embedding.

The first layer performs input projection, mapping a scalar to a higher-dimensional space (64). The second layer serves as a hidden layer, while the final layer aligns the features to match the spatial dimensions of the encoder’s bottleneck output. Being only a few layers, the MLP introduces minimal computational overhead while effectively capturing the influence of peak brightness on the HDR reconstruction process. The entire module adds approximately 33,000 parameters to the model, representing a negligible increase over the original 54M-parameter architecture proposed by Endo et al. [3]. To summarize, the module receives a single scalar value, P_sample_, and learns a rich feature representation of channels aligned with the size of the bracketed exposure bottleneck. The design results in a feature vector:

(3) $$Z_{cond}=MLP\left(P_{sample}\right),\;Z_{cond}\in R^{512}$$

The network’s bracketed exposure generator also encodes the SDR image in Z_img_. Expansion must be made in accordance with both. We employ a spatially broadcast additive injection strategy. Since Z_img_ is a 3D tensor of shape H’ × W’ × 512 (representing spatial features) and Z_cond_ is a 1D vector of size 512 (representing the global feature), dimensions must be aligned before fusion. We first unsqueeze and broadcast to a Z’_cond_ which spatially matches the encoder output’s height and width.

(4) $${Z^{\prime }}_{cond}=Z_{cond}.unsqueeze\left(-1\right).unsqueeze\left(-1\right),{Z^{\prime }}_{cond}\in R^{512,1,1}$$

The fusion is then performed via element-wise addition:

(5) $$Z_{display-aware}\left(x,y\right)=Z_{img}\left(x,y\right)+{Z^{\prime }}_{cond}$$

Equation (5) shows the addition of the global brightness bias to every spatial location in the feature map. Adding the information to every channel structurally links image encoding with the display information. After passing through subsequent activations, the brightness embedding modulates the magnitude of existing features rather than introducing separate channels that might not link in a coherent manner.

Standard U-Net designs are great at performing generic mappings between input and output. However, they lack the ability to adapt the overall reconstruction to external conditions. In our case, the same stack of bracketed exposures would be generated regardless of the target display’s peak brightness. The importance of adaptation in tone mapping is underlined by the real-time, display-aware approach of Eilertsen et al. [14], which demonstrates that display-adaptive strategies consistently surpass the performance of static operators. Research targeting the U-Net architecture found significant benefits in conditioning the learning process. Feature-wise linear modulation (FiLM), introduced by Perez et al. [23], shows that combining a network’s latent space by injecting it into other layers can significantly improve performance across various tasks. Tunable U-Nets, proposed by Kang et al. [24], demonstrated the concept of modulating the U-Net’s outputs using a scalar external parameter. Their example was image binarization, where the threshold value is essential, and retraining would be needed if it were to change. Similarly, in the audio domain, conditioned U-Nets have used auxiliary vectors to dynamically control source separation tasks, as done by Cohen-Hadria et al. [25].

We demonstrated that this approach is highly effective for reverse tone mapping. Our work most closely relates to Kang et al. [24] and lays the foundation for future research in display-aware HDR processing. So far, this device-aware philosophy has been largely absent in this field. As stated before, Luzardo et al. [15] propose a display-adaptive pipeline for video content, but their approach relies on mathematical transfer functions and a heuristic feature-detection method. In contrast, our method integrates display adaptation directly into the deep feature space, enabling guided reconstruction. Tuning the parameter adapts the result with no effort and keeps our solution current with hardware display evolution.

### 3.3. Training Procedure

All other deep-based rTM solutions rely on static datasets for training. Such models are limited to the distribution of luminance observed by the network and thus learn to reconstruct HDR images with a fixed peak brightness (the one prevalent in the training set). A more versatile model should be able to adapt to a range of HDR monitor specifications. To overcome this limitation, we propose a pipeline that adapts the label data dynamically during training.

#### 3.3.1. Dataset Preparation

We acquire images from the Polyhaven HDR dataset [26]. This database offers a wide variety of real-world scenes, ranging from complex indoor lighting setups to expansive outdoor environments. Data collection was done with professional cameras and in high resolution and quality. LuxDIT, proposed by Liang et al. [27], highlighted the importance of such a diverse dataset for testing their HDR environment map reconstruction method. In EdgeRelight360, by Lin et al. [28], Polyhaven is used as the main source of HDR data for training. Our dataset consists of 900 high-definition images. The EXR format, introduced by Industrial Light & Magic, is used to store the HDR representation. 4K resolution and 32-bit floating precision per channel images, capable of representing up to 24 stops of dynamic range, preserve details in the brightest highlights and deepest shadows simultaneously. The dataset is split into 80% for training, 10% for validation, and 10% for testing, ensuring a diverse range of scenes in each subset.

Selection was made using a random sampling strategy to ensure a wide variety of scenes and lighting conditions are represented. To achieve even luminance levels across each set, scenes are stratified by their dynamic range, $EV_{scene}$. In this manner, both high- and low-contrast settings are present in each one. Training is performed on 512 × 512 resized versions. No random cropping or augmentation is applied.

As stated above, we adopt Endo et al.’s [3] approach to infer bracketed exposures from our model. Hence, the next step is to generate bracketed exposures from each HDR image in the dataset. Debevec and Malik [29] correlated the image results at a given exposure level based on the scene’s radiance map. The following equation converts the entire EXR image into multiple SDR images, each representing a specific exposure level:

(6) $$Z_{i,j}=f\left(E_{i}\cdot \Delta t_{j}\right),$$

where Z_i,j_ is the synthesized SDR pixel value; f: HDR → LDR is the camera response function (CRF); E_i_ is the HDR irradiance value map at pixel i; $\Delta t_{j}$ is the exposure duration for the j-th bracketed image.

Before fetching the images from the HDR base, we first assess its overall brightness difference in logarithmic light stops:

(7) $$EV_{scene}=log_{2}\frac{L_{max}}{\left(L_{min}+\varepsilon \right)},$$

where $L_{max}$ and $L_{min}$ are the maximum and minimum luminance values of the HDR image.

This gives a scene-dependent range, guiding the overall capabilities of the image to avoid enhancing it too much on a capable display.

Then, a physical representation of a camera should be used to create images. To apply the non-linear response, we randomly select representative camera response functions (CRFs) from the DoRF database, created by Grossberg and Nayar [30]. As in Endo et al. [3], K-means clustering is used to select 5 representative curves. Utilizing different CRFs across the training set, we force the network to learn a generalized inverse mapping that is robust to different sensor characteristics, rather than overfitting to a specific one. Finally, the synthesized pixel values are clipped to [0, 1] and quantized to 8-bit standard precision (0–255 integer values). This mimics the information loss inherent in standard digital photography formats, such as JPEG.

Given the availability of the camera response functions (CRFs) and the source HDR images, it is sufficient to vary the exposure times. Accordingly, we generated *N* = 9 images at different exposure levels. To correctly adjust for display capabilities without introducing physical inaccuracies, we directly determine the target 9 fractional exposure times:

(8) $$∆t_{j}=\tau^{j}j\;\in \{-4,\ldots ,4\}$$

The exposure stop, τ, is computed based on the peak brightness and the overall brightness of the input image, as shown in (14). In this approach, the sequence of images gradually varies from underexposed to overexposed. The input HDR image is normalized to ensure that the middle exposure, after applying the CRF, corresponds to a mid-grey value with a geometric mean of 0.5.

#### 3.3.2. Dynamic Display Adaptation

During training, we dynamically sample a targeted peak brightness ($P_{sample}$). Based on this one and the already computed image brightness ($EV_{scene}$), we compute the optimal exposure stop.

We implemented a log-uniform sampling strategy to select the target peak brightness, $P_{sample}$, for each training iteration. This approach ensures model generalization across a wide range of display capabilities, from low-end office monitors (around 100 nits) to high-end, studio-like displays (reaching even 4000 nits).

Two bounds are chosen based on current HDR monitor technology:

(9) $$P_{min}\;=\;100\;nits,\;P_{max}\;=\;4000\;nits\;$$

To align with the Weber–Fechner law of perception, the sampling is performed in the logarithmic space:

(10) $$log\left(P_{\mathrm{sample}}\right)∼Uniform\left(log\left(P_{min}\right),\;log\left(P_{max}\right)\right)\;$$

This ensures equal representation of all brightness levels during training, preventing the model from overfitting to a specific range of capabilities. We must note that, as HDRs evolve, the limits can be updated accordingly without changing the training procedure.

At training time, we generate N = 9 bracketed exposures. This process is guided by the target display parameter, $P_{sample}$, and capped by the scene’s actual dynamic range, $EV_{scene}$.

We begin by defining two baseline constants, each grounded in prior work:

(11) $$P_{baseline}=250\;nits,EV_{baseline}=4.0\;EV$$

We further compute the display capability regarding our method by mapping $P_{sample}$ to our maximum targeted exposure range as follows:

(12) $$EV_{display}=EV_{baseline}+{log}_{2}\left(\frac{P_{sample}}{P_{baseline}}\right)$$

Previous works, such as Endo et al. [3], utilized a fixed 9-image stack with the same spacing of τ = √2, which resulted in a strict 4 EV coverage for all HDR reconstructions. By mapping our 250-nit $P_{baseline}$ to this 4 $EV_{baseline}$ span, we establish that our model’s capability matches the fixed output of prior methods. Consequently, as the target display scales to true HDR luminance levels, our method dynamically expands the requested exposure range to 6 or 8 EV. This enables the network to predict significantly broader tonal ranges and extract highlight details that fixed-stack methods inherently overlook.

However, unconditionally expanding the exposure range based solely on display hardware introduces an important pitfall: it risks artificially stretching naturally low-contrast scenes, forcing the network to hallucinate non-existent contrast. To prevent this, we also strictly bound the requested display capability by the physical reality of the ground-truth HDR image, $EV_{scene}$. The final exposure range is therefore:

(13) $$EV_{final}=min\left(EV_{scene},EV_{display}\right)$$

After we know the final exposure value in stops, we can compute the final τ, so that the range is uniformly spanned, as shown in Table 1. Each step must advance by exactly $\frac{EV_{final}}{8}$ stops. Therefore, we define our dynamic exposure multiplier τ as:

(14) $$\tau =2^{\left(\frac{EV_{final}}{8}\right)}$$

We further calculate the exact exposure time multiplier for each frame index as in (8). Table 2 presents the combined $EV_{final}$ calculation. Low-contrast scenes are not overly extended on capable devices, being capped by the actual range. In contrast, high-range ones are expanded up to the device limit, enabling full utilization.

### 3.4. Actual Training

We implemented the above-described model using the PyTorch 2.9.0 framework. Training was conducted on the National University of Science and Technology’s Automatic Control and Computer Science Department’s cluster, using NVIDIA A100 GPUs. Unlike the solution proposed by Endo et al. [3], which utilized a batch size of one, the high VRAM capacity of the A100 GPUs allowed us to train with a batch size of 8, significantly stabilizing the gradient estimation. Moreover, as discussed by Ioffe and Szegedy [17], batch normalization relies on batch statistics, and using smaller batches results in noise and inaccurate results. We utilized the Adam optimizer [31] with a fixed learning rate of 0.0002 and momentum terms β_1_ = 0.5 and β_2_ = 0.999. The weights of the 2D encoder and 3D decoder were initialized using zero-mean Gaussian noise with a standard deviation of 0.02, following the protocol of Endo et al. [3]. In deep learning environments, initial weights are a key factor, along with activation function strategy. Xavier initialization preserves variance across layers by equating input and output variance, making it well-suited to symmetric activations such as those found in the sigmoid family. He Normal extends this analysis to account for the variance reduction that ReLU introduces in both forward and backward propagation, making it better suited to ReLU-activated layers and known to converge faster in deep networks. A mixed strategy using He Normal for the intermediate convolutional and deconvolutional layers and Xavier for the sigmoid output layer would therefore be theoretically more motivated than our uniform Gaussian scheme. However, following Endo et al. [3], our simpler approach empirically stabilizes early training in multi-exposure encoder–decoder architectures by starting the network in a near-linear regime. A comparison of these initialization strategies remains a direction for future work.

We trained for 100 epochs in total. The entire process took about 40 h. Checkpointing was performed every 5 epochs to allow comparison of intermediate results and selection of the best-performing model based on validation metrics.

As a loss function, we employ a simple L1 reconstruction loss over the entire 9-exposure bracketed stack:

(15) $$L=\frac{1}{\left(N\cdot H\cdot W\right)}\sum \sum ||(S_{pred}^{i}(x,y)-S_{GT}^{i}(x,y)|{|}_{1},$$

where N = 9 is the number of exposures and $S^{i}$ represents the i-th exposure in the stack. The loss function does not need to consider the sampled display brightness because this has already been done by adjusting the stack of images. The dense 9-exposure stack is generated via actual exposure time variation, as described, by applying the same camera response function to differently scaled HDR radiance. This embeds physical relationships: higher exposures must be brighter, maintain color consistency, and preserve spatial structure. Hence, the reconstruction task itself changes with brightness, forcing the network to learn display-specific inverse camera response functions rather than a universal one, followed by gain adjustment.

### 3.5. Final Image Formation

The proposed display-aware model outputs bracketed SDR images, which act as an HDR representation. From this, we have the options to directly reconstruct the HDR or obtain a display-ready tone-mapped image.

Firstly, for explicit HDR reconstruction, the Debevec and Malik method [29] can be used. This reverse process was previously implemented for processing our training database, as stated in Section 3.3.1. Having the original, high-precision radiance map in the form of an EXR file, the Debevec and Malik method was used, based on already known film response functions (CRFs). The same approach can also be used for constructing the final image based on the model’s output. Middle exposure time can be estimated. Subsequently, for each bracketed image, the exposure stop value τ is used to compute it. The resulting HDR image can be used for further processing, such as tone mapping or quality evaluation.

Secondly, for a display-ready image, we can use a tone-mapped view. Mertens et al. [16] introduced a method for visualizing a high-quality representation of the HDR image without reconstructing it. Their approach fuses the bracketed exposure images into a tone-mapped representation, suitable for display on any monitor. A weighted average of the input images is computed based on contrast, saturation, and exposure level, generating a tone-mapped representation that naturally combines the display-aware luminance distribution encoded in the predicted stack. The result is a single, display-ready image that reflects both the enhanced dynamic range and the target screen’s brightness value, requiring no further processing before displaying. Furthermore, certain standard web browsers struggle to display actual HDR imaging, but they can easily render the fused output from Mertens et al.’s method. The visualizations presented below were created from the bracketed exposures inferred, using this technique.

## 4. Results

### 4.1. Testing Interface

To facilitate qualitative inspection and interactive testing, we developed a web-based application built using Gradio, introduced by Abid et al. [32]. The interface allows users to upload an SDR image and select either a preset or custom peak brightness value. Eight display presets are provided, offering common display categories: SDR monitors (250 nits), entry-level HDR (400, 600 nits), mid-range HDR (1000 nits), and high-end studio monitors (4000 nits). Upon processing, the application visualizes the generated stack of display-conditioned exposures alongside an optional HDR reconstruction and tone-mapped preview.

This tool was used throughout our experiments to quickly compare the model’s output across different target displays and to validate that the conditioning on peak value produces intuitively consistent changes in local contrast and highlight rendering.

All inference experiments were conducted on a single NVIDIA RTX 2060 GPU. The model can process a 512 × 512 SDR input and produce the complete nine-exposure bracketed stack in under one second, thereby enabling interactive operation within the testing interface.

The final view is provided using Mertens et al. [16], as stated above. It constitutes a tone-mapped representation directly viewable on any monitor. We also made it possible to reconstruct using Debevec and Malik’s [29] method. The true generated EXR file can be downloaded and examined on proper hardware.

### 4.2. Evaluation

We assessed the performance of our display-aware rTM model using a four-part approach: visual inspection of the outputs, quantitative brightness-distribution metrics, pixel-level qualitative metrics for the true HDR reconstruction, and perceptual quality evaluation using a display-based metric. Testing was done using a set of unseen HDR images from the Polyhaven dataset, classical inputs from the rTM literature such as those used by Endo et al. [3], and real SDR images from the Div2K dataset [33].

The experimental comparison is intentionally focused on DrTMO as the architectural backbone of the proposed method. The primary goal of our work is to demonstrate that conditioning the rTM process on target display peak brightness is a meaningful and measurable improvement over a display-agnostic equivalent. Recent advances, including diffusion-based and Transformer-based architectures, are display-agnostic by design and do not share architectural patterns with our solution. Investigating how display awareness can be integrated into such architectures, and whether the perceptual gains demonstrated here scale with model capacity, is a primary direction for future work.

#### 4.2.1. Visual Inspection

**Figure 3:**
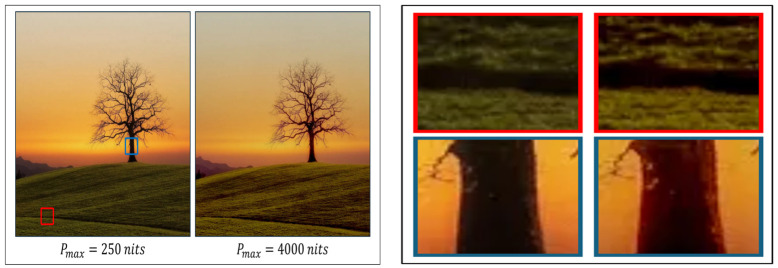
Output comparison for a sunset scene at 250 nits (**left**) and 4000 nits (**right**). Red and blue crops show close-up views from the grass and the tree trunk regions.

Adjusting the target peak brightness yielded notably better outcomes than baseline-agnostic solutions. As expected, lower values produced more conservative HDR reconstructions, with reduced highlight emphasis and overall contrast. Conversely, higher peak brightness capable settings resulted in vivid highlights and enhanced local contrast, effectively utilizing the expanded dynamic range. The model demonstrated strong performance in understanding how to allocate luminance across the image based on the target display’s capabilities. Furthermore, detailed close-up examination shows that the model successfully reconstructs details in challenging regions, based on the capabilities of the target display. Figure 3 illustrates both aspects.

**Figure 4:**
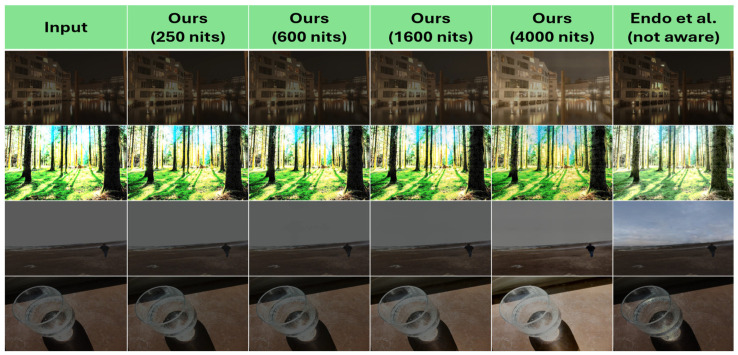
Visual comparison of our display-aware method at four target peak brightness levels (250, 600, 1600, 4000 nits) against the display-agnostic Endo et al. [3] baseline across four diverse scenes.

We further visually evaluate our display-aware model against the baseline DrTMO by Endo et al. [3] across various test scenes at four selected peak brightness levels (250, 600, 1600, 4000 nits). Figure 4 shows the graphical visual comparison. The baseline produces a single fixed output regardless of target display. Our pipeline demonstrates successful display-aware understanding.

The above evaluations were done using SDR images synthesized from HDRs, from our testing Polyhaven database. To provide a more accurate representation of our work, we conducted further testing on 20 real SDR images collected from the Div2K dataset. The model visibly enhances local contrast on all nit values. This highlights the fact that the adjusted generated stack contains gradually more details with brightness levels, and Mertens fusion can successfully integrate them. For images with interior lighting, the network recovers visible details in highlight and shadow areas that are compressed in the input SDR. For exterior scenes the difference from the input is smaller, which is the conservative behavior intended, resulting in scenes with reduced dynamic range which do not benefit substantially from an aggressive HDR reconstruction.

#### 4.2.2. Extreme Lighting Conditions

**Figure 5:**
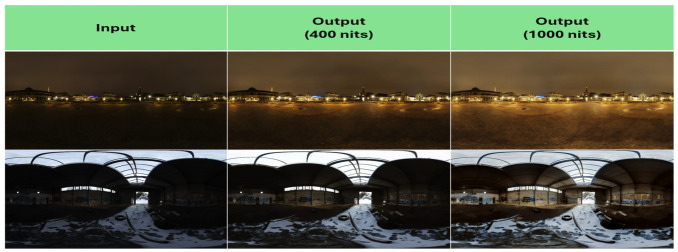
Low-light model reconstruction result for two challenging scenes at selected peak brightness values of 400 and 1000 nits.

We evaluate our model on challenging reconstruction tasks involving extremely low-light environments. Specifically, we assess performance on two demanding low-light SDR inputs: a night-time outdoor city with nearly no recoverable shadow detail, and a high-contrast indoor scene where most of the image is underexposed. Figure 5 shows the results. In the night city, the model can recover architectural structure and lighting patterns, with no observable blurring in the reconstructed mid-tones. The luminance boost scales effectively with the target peak brightness, allowing higher nit settings to progressively reveal more foreground detail. In the high-contrast indoor scene, the model reconstructs wall texture and spatial structure from the deeply shadowed regions while preserving the bright exterior opening. In both cases, sharpness is consistently maintained in the reconstructed areas, indicating that the predicted exposure stack provides sufficient gradient information even under severe underexposure. Overall, these results show that the model does not cause systematic blurring in low-light conditions. However, the recovery of fine texture in near-black regions remains limited by the lack of recoverable signal in the input.

#### 4.2.3. Quantitative: Brightness Distribution

We analyzed how the brightness is structurally distributed across the tone-mapped outputs of the Div2K real-SDR set. To quantitatively calculate, we measured the luminance usage of the final outputs. The metrics computed regarding luminance are as follows: mean luminance, peak luminance (computed from the top 1% values), and display peak utilization ratio.

(16) $$Mean\;luminance:\bar{L}=\frac{1}{N}\sum_{i=1}^{N}L_{i}$$

(17) $$Peak\;brightness:{L_{p}}^{top1}=top_{1\%}\left(sort\left(\vec{L}\right)\right)$$

(18) $$Peak\;utilization\;ratio:PU=\left(\frac{{L_{p}}^{top1}}{P_{target}}\right)\times 100$$

Table 3 contains the computed values. It is obvious that we obtained better, targeted results than the non-display-aware version. DrTMO produces fixed luminance distribution regardless of the target display’s peak brightness (175 nits mean luminance and 1342 nits peak), resulting in severe overexposure when targeting low-brightness displays (537% peak utilization at 250 nits) and underutilization in high-brightness displays (34% at 4000 nits). In contrast, our solution achieves better overall usage for the selected HDR settings, as shown by the utilization ratio.

#### 4.2.4. Qualitative Evaluation: True HDR Results

To evaluate the quality of the reconstructed HDR content directly, we compute two pixel-level metrics on the HDR images generated using the Debevec and Malik reconstruction [29]: local contrast (measured as the average local standard deviation over the image) and colorfulness (Hasler–Süsstrunk score). These complement the luminance distribution analysis by capturing structural and chromatic richness in the reconstructed HDR rather than its display-level brightness allocation. The average metrics are presented in Table 4.

The proposed display-aware approach achieves higher local contrast and colorfulness across all target peak brightness levels (250–4000 nits) compared to the baseline DrTMO, which remains agnostic to the display. Table 5 gives a more image-specific metrics breakdown. It can be observed that we frequently achieve higher local contrast and colorfulness scores compared to targets with higher peak brightness. This result occurs because, at lower peak brightnesses, the model is pushed to compress the newly hallucinated dynamic range into a much narrower final luminance band. To preserve visibility and structural detail within this constrained range, the network aggressively boosts mid-tones and local variations, artificially strengthening the local contrast and color saturation.

#### 4.2.5. Perceptual Quality Evaluation: HDR-VDP-3

To complement the luminance distribution and pixel-level metrics, we evaluate perceptual quality using HDR-VDP-3 [34], a display-referred metric that models the human visual system’s response to luminance and explicitly accounts for the target display’s peak brightness. This makes it particularly well-suited to evaluating display-aware reconstruction, showcasing how well the viewing experience matches the specific screen for which the image was generated. We use the publicly available HDR-VDP-3 implementation with default visual model parameters, setting the display peak luminance parameter to match each tested brightness level (250, 600, 1600, and 4000 nits) and a pixel-per-degree value of 30, corresponding to a typical viewing distance for a desktop HDR monitor. For each of the 90 test images from the Polyhaven dataset, we reconstruct an HDR image using both our method and the DrTMO baseline, following Debevec and Malik [29], and compute HDR-VDP-3 scores against the ground-truth HDR at each target peak brightness level. Table 6 summarizes the results.

At 250 and 600 nits, our method achieves mean HDR-VDP-3 scores of 4.16 and 4.31, respectively, compared to 3.94 and 4.17 for the baseline, and wins on 90% and 100% of test images. These gains reflect the baseline’s systematic overexposure at low brightness targets, which HDR-VDP-3 penalizes as visible distortion relative to the reference. At 1600 nits, the baseline achieves a marginally higher mean score (5.21 vs. 4.82), winning on 65% of images. This result is explained by the fact that the baseline’s fixed implicit luminance output is closest to the 1600 nit target among all tested levels, accidentally aligning with that display’s range without any conditioning. Importantly, this is the only brightness level at which a display-agnostic model achieves competitive performance, and this occurs solely because its fixed output fortuitously aligns with the characteristics of that particular target. We argue that a display-agnostic model produces the best reconstructions only at the single brightness level that matches its training distribution. At 4000 nits, our method recovers strongly, achieving a mean score of 6.67 versus 4.90 for the baseline and wins on 90% of images. This is the largest absolute gap across all tested levels, reflecting our method’s ability to correctly expand the dynamic range to fill the display’s capability, while the baseline leaves most of it unused. Taken together, these results demonstrate that display-aware conditioning provides consistent perceptual benefits across the practical HDR display range, except in the narrow band where the baseline’s implicit output incidentally matches the target display.

## 5. Conclusions

Our results show that incorporating display awareness into the SDR-to-HDR conversion process enables tailored reconstructions that better leverage each target display’s capabilities, yielding more visually compelling results. Conditioning the model on the display’s peak brightness allows it to capture the relationship between display characteristics and optimal luminance distribution. By targeting the reconstruction to a concrete physical constraint, our approach addresses the “one-to-many” mapping challenge in reverse tone mapping and achieves improved adaptation across a broad spectrum of display types. This work creates a foundation for future studies in display-aware HDR processing, opening the path for integrating additional display parameters and investigating new conditioning strategies to improve the model’s capacity and effectiveness.

## 6. Limitations

Despite the positive results, our approach has several key limitations. Firstly, our training labels are generated using a deterministic function. Specifically, we computed varying exposure times based on the target display’s peak brightness and generated the corresponding SDR image stacks from the HDR ground truth using the Debevec and Malik method. While this effectively approximates the required luminance scaling, an ideal approach would involve perceptual studies to determine the most visually pleasing, optimal HDR representation tailored specifically to each target display’s characteristics, as described in future work. Furthermore, as noted in our evaluation, the absence of extensive subjective user studies conducted on fully calibrated HDR monitors means that our current quality assessment relies heavily on objective representative metrics.

Secondly, our current model architecture is based on U-Net, which may not fully capture the complex relations between display characteristics and SDR image content. More advanced architectures could potentially enrich the model’s ability to learn subtle relationships between the input content and the target display’s capabilities.

## 7. Future Work

We demonstrated that our initial hypothesis is correct: integrating peak brightness conditioning into the SDR-to-HDR conversion process allows the model to learn a meaningful correlation between display capabilities and appropriate luminance distribution.

However, our work is only a first step in this direction. More parameters can be integrated into the model, such as color gamut, contrast ratio, black levels, and type of display technology (OLED, LCD, microLED), enabling even more targeted training.

The current architecture is based on a U-Net backbone. Future research could integrate display awareness in alternative architectures, such as Transformers. Forms of more sophisticated conditioning mechanisms, like attention-based fusion introduced by Dai et al. [35], could enhance the model’s ability to adapt to complex display characteristics. Extending the display-aware conditioning principle to such more recent architectures represents an impactful direction for future research, as the gains demonstrated here suggest that display awareness is a broadly applicable and architecturally non-dependent improvement.

The final learning label could be further enhanced by incorporating perceptual studies aimed at identifying the most visually appealing HDR representation for each target display. While our current method relies on a deterministic function to fuse the target stack of exposures, a more robust solution would integrate human-in-the-loop optimization to discover HDR representations that maximize perceived quality across different display types. In the future, we intend to conduct extensive subjective user studies on fully calibrated HDR monitors to both validate the perceptual advantages of our display-aware approach and create a dataset of user preferences for HDR representations across different display capabilities.

## Figures and Tables

**Table 1:** Exposure multiplier values for reference peak brightness levels.

Brightness (nits)	EV	$\tau_{effective}$
250	4	√2 ≈ 1.414
1000	6	1.68
4000	8	2.0

**Table 2:** Combined calculation for different scenes and display scenarios.

Scene	$P_{sample}$	$EV_{scene}$	$EV_{display}$	$EV_{final}$	Task
Foggy day	4000 nit	3 EV	8 EV	3 EV	Narrow
Sunset	4000 nit	8 EV	8 EV	8 EV	Wide
Foggy day	250 nit	3 EV	4 EV	3 EV	Narrow
Sunset	250 nit	8 EV	4 EV	4 EV	Medium

**Table 3:** Mean luminance, peak luminance, and peak utilization ratio at four target peak brightness levels comparing our solution against the display-agnostic DrTMO baseline on the Div2K real-SDR test set.

Target Peak Brightness (Nits)	Model	Mean Luminance (Nits)	Peak Luminance (Nits)	Overall Ratio
250	Endo et al. (DrTMO)	175.2 ± 32.1	1342.3 ± 1128.5	537%
	Display-Aware (Ours)	110.5 ± 26.8	485.2 ± 378.4	194%
	Relative Change	−37%	−64%	2.8× better
600	Endo et al. (DrTMO)	175.2 ± 32.1	1342.3 ± 1128.5	224%
	Display-Aware (Ours)	125.3 ± 28.2	712.8 ± 615.3	119%
	Relative Change	−28%	−47%	1.9× better
1600	Endo et al. (DrTMO)	175.2 ± 32.1	1342.3 ± 1128.5	84%
	Display-Aware (Ours)	188.7 ± 33.5	1524.8 ± 1087.3	95%
	Relative Change	+8%	+14%	1.13× better
4000	Endo et al. (DrTMO)	175.2 ± 32.1	1342.3 ± 1128.5	34%
	Display-Aware (Ours)	231.5 ± 47.2	1965.4 ± 998.6	49%
	Relative Change	+32%	+46%	1.44× better

**Table 4:** Overall computed average metrics for the selected subset of images across four target peak brightness levels.

Metric	Baseline (DrTMO)	Ours (250 nits)	Ours (600 nits)	Ours (1600 nits)	Ours (4000 nits)
Local Contrast	6.26	7.20	7.37	7.08	6.63
Colorfulness	47.02	64.09	62.35	54.88	49.81

**Table 5:** Per-image local contrast and colorfulness scores across four target peak brightness levels for selected images from the Div2K real-SDR set.

Image	Metric	Baseline (DrTMO)	Ours (250 nits)	Ours (600 nits)	Ours (1600 nits)	Ours (4000 nits)
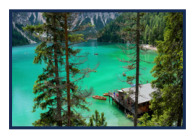	Local Contrast	11.11	12.42	12.66	13.15	12.32
	Colorfulness	65.42	86.39	84.74	77.16	67.52
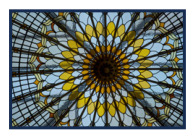	Local Contrast	10.66	12.26	12.59	12.00	11.53
	Colorfulness	53.45	68.33	65.25	57.46	54.22
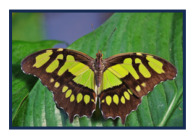	Local Contrast	4.98	5.32	5.39	5.23	5.32
	Colorfulness	50.49	73.09	70.42	62.91	54.61
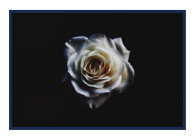	Local Contrast	1.12	2.47	2.57	1.82	2.22
	Colorfulness	12.99	21.48	22.14	22.78	20.04
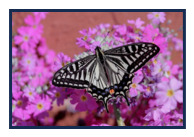	Local Contrast	3.41	3.54	3.62	3.65	3.78
	Colorfulness	52.76	61.15	59.20	54.08	53.65

**Table 6:** HDR-VDP-3 perceptual quality scores comparing our solution against the display-agnostic DrTMO baseline across four target peak brightness levels on 90 Polyhaven test images.

Target Peak Brightness (nits)	Ours Mean	Baseline Mean	Difference (Ours–Base)	Ours (Win Rate)
250	4.16	3.94	+0.22	90%
600	4.31	4.17	+0.14	100%
1600	4.82	5.21	−0.39	35%
4000	6.67	4.90	+1.77	90%
All	4.51	4.24	+0.27	78%

## Data Availability

The original contributions presented in this study are included in the article. Further inquiries can be directed to the corresponding author.

## References

[1] Chen K., Matsuda N., McElvain J., Zhao Y., Wan T., Sun Q., Chapiro A. (2025). What Is HDR? Perceptual Impact of Luminance and Contrast in Immersive Displays. Proceedings of the Special Interest Group on Computer Graphics and Interactive Techniques Conference Conference Papers, Vancouver, BC, Canada, 10–14 August 2025.

[2] Hammou D., Krasula L., Bampis C.G., Li Z., Mantiuk R.K. The Effect of Viewing Distance and Display Peak Luminance—HDR AV1 Video Streaming Quality Dataset. Proceedings of the 2024 16th International Conference on Quality of Multimedia Experience (QoMEX).

[3] Endo Y., Kanamori Y., Mitani J. (2017). Deep Reverse Tone Mapping. ACM Trans. Graph..

[4] Banterle F., Ledda P., Debattista K., Chalmers A. (2006). Inverse Tone Mapping. Proceedings of the 4th International Conference on Computer Graphics and Interactive Techniques in Australasia and Southeast Asia, Kuala Lumpur, Malaysia, 29 November–2 December 2006.

[5] Akyüz A.O., Fleming R., Riecke B.E., Reinhard E., Bülthoff H.H. (2007). Do HDR Displays Support LDR Content? A Psychophysical Evaluation. ACM Trans. Graph..

[6] Eilertsen G., Kronander J., Denes G., Mantiuk R.K., Unger J. (2017). HDR Image Reconstruction from a Single Exposure Using Deep CNNs. ACM Trans. Graph..

[7] Cai J., Gu S., Zhang L. (2018). Learning a Deep Single Image Contrast Enhancer from Multi-Exposure Images. IEEE Trans. Image Process..

[8] Martins G.d.L., Lopez-Cabrejos J., Martins J., Leher Q., Ferreti G.d.S., Carvalho L.H.C., Lima F.B., Paixão T., Alvarez A.B. (2025). A Review Toward Deep Learning for High Dynamic Range Reconstruction. Appl. Sci..

[9] Bemana M., Leimkühler T., Myszkowski K., Seidel H.-P., Ritschel T. (2024). Bracket Diffusion: HDR Image Generation by Consistent LDR Denoising. arXiv.

[10] Goswami A., Singh A.R., Banterle F., Debattista K., Bashford-Rogers T. (2025). Semantic Aware Diffusion Inverse Tone Mapping. J. Phys. Conf. Ser..

[11] Reinhard E., Stark M., Shirley P., Ferwerda J. (2002). Photographic Tone Reproduction for Digital Images. ACM Trans. Graph..

[12] Drago F., Myszkowski K., Annen T., Chiba N. (2003). Adaptive Logarithmic Mapping For Displaying High Contrast Scenes. Comput. Graph. Forum.

[13] Mantiuk R., Daly S., Kerofsky L. (2008). Display Adaptive Tone Mapping. ACM Trans. Graph..

[14] Eilertsen G., Mantiuk R.K., Unger J. (2015). Real-Time Noise-Aware Tone Mapping. ACM Trans. Graph..

[15] Luzardo G., Kumcu A., Aelterman J., Luong H., Ochoa D., Philips W. (2024). A Display-Adaptive Pipeline for Dynamic Range Expansion of Standard Dynamic Range Video Content. Appl. Sci..

[16] Mertens T., Kautz J., Van Reeth F. Exposure Fusion. Proceedings of the 15th Pacific Conference on Computer Graphics and Applications (PG’07).

[17] Ioffe S., Szegedy C. (2015). Batch Normalization: Accelerating Deep Network Training by Reducing Internal Covariate Shift. arXiv.

[18] Maas A.L., Hannun A.Y., Ng A.Y. Rectifier Nonlinearities Improve Neural Network Acoustic Models. Proceedings of the ICML Workshop on Deep Learning for Audio, Speech, and Language Processing.

[19] Brock A., Lim T., Ritchie J.M., Weston N. (2016). Generative and Discriminative Voxel Modeling with Convolutional Neural Networks. arXiv.

[20] Vondrick C., Pirsiavash H., Torralba A. (2016). Generating Videos with Scene Dynamics. Proceedings of the 30th International Conference on Neural Information Processing Systems, Barcelona, Spain, 5–10 December 2016.

[21] He K., Zhang X., Ren S., Sun J. Deep Residual Learning for Image Recognition. Proceedings of the 2016 IEEE Conference on Computer Vision and Pattern Recognition (CVPR).

[22] Wang C., Banterle F., Ren B., Timofte R., Lu X., Peng Y., Ge C., Sun Z., Zhou Z., Li Z. AIM 2025 Challenge on Inverse Tone Mapping Report: Methods and Results. Proceedings of the 2025 IEEE/CVF International Conference on Computer Vision Workshops (ICCVW).

[23] Perez E., Strub F., de Vries H., Dumoulin V., Courville A. (2017). FiLM: Visual Reasoning with a General Conditioning Layer. arXiv.

[24] Kang S., Uchida S., Iwana B.K. (2021). Tunable U-Net: Controlling Image-to-Image Outputs Using a Tunable Scalar Value. IEEE Access.

[25] Cohen-Hadria A., Roebel A., Peeters G. Improving Singing Voice Separation Using Deep U-Net and Wave-U-Net with Data Augmentation. Proceedings of the 2019 27th European Signal Processing Conference (EUSIPCO).

[26] Polyhaven HDR Dataset. https://polyhaven.com/hdris.

[27] Liang R., He K., Gojcic Z., Gilitschenski I., Fidler S., Vijaykumar N., Wang Z. (2025). LuxDiT: Lighting Estimation with Video Diffusion Transformer. arXiv.

[28] Lin M.-H., Reddy M., Berger G., Sarkis M., Porikli F., Bi N. EdgeRelight360: Text-Conditioned 360-Degree HDR Image Generation for Real-Time On-Device Video Portrait Relighting. Proceedings of the 2024 IEEE/CVF Conference on Computer Vision and Pattern Recognition Workshops (CVPRW).

[29] Debevec P.E., Malik J., Whitton M.C. (2023). Recovering High Dynamic Range Radiance Maps from Photographs. Seminal Graphics Papers: Pushing the Boundaries.

[30] Grossberg M.D., Nayar S.K. (2003). Determining the Camera Response from Images: What Is Knowable?. IEEE Trans. Pattern Anal. Mach. Intell..

[31] Kingma D.P., Ba J. (2017). Adam: A Method for Stochastic Optimization. arXiv.

[32] Abid A., Abdalla A., Abid A., Khan D., Alfozan A., Zou J. (2019). Gradio: Hassle-Free Sharing and Testing of ML Models in the Wild. arXiv.

[33] Timofte R., Agustsson E., Gool L.V., Yang M.-H., Zhang L., Lim B., Son S., Kim H., Nah S., Lee K.M. NTIRE 2017 Challenge on Single Image Super-Resolution: Methods and Results. Proceedings of the 2017 IEEE Conference on Computer Vision and Pattern Recognition Workshops (CVPRW).

[34] Mantiuk R.K., Hammou D., Hanji P. (2023). HDR-VDP-3: A multi-metric for predicting image differences, quality and contrast distortions in high dynamic range and regular content. arXiv.

[35] Dai Y., Gieseke F., Oehmcke S., Wu Y., Barnard K. Attentional Feature Fusion. Proceedings of the 2021 IEEE Winter Conference on Applications of Computer Vision (WACV).

